# Präzisionsmedizin in der Kinderneurologie am Beispiel der neuen Therapien

**DOI:** 10.1007/s00115-021-01251-5

**Published:** 2022-01-17

**Authors:** Andreas Ziegler

**Affiliations:** Zentrum für Kinder- und Jugendmedizin, Sektion für Neuropädiatrie und Stoffwechselmedizin, Universitätsklinikum Heidelberg, Im Neuenheimer Feld 430, 69120 Heidelberg, Deutschland

**Keywords:** Personalisierte Medizin, Individualisierte Medizin, Gentherapie, Neuromuskuläre Erkrankungen, Neugeborenenscreening, Personalized medicine, Individualized medicine, Gene therapy, Neuromuscular diseases, Neonatal screening

## Abstract

**Hintergrund:**

In den letzten Jahren haben sich die Möglichkeiten der molekularen Diagnostik und Therapie seltener Erkrankungen im Kindesalter stark verbessert. Erste genmodifizierende Arzneimittel wurden inzwischen zugelassen, sie leiten eine neue Ära der Präzisionstherapie in der Kinderneurologie ein.

**Ziele:**

Dieser Beitrag beschreibt die dynamischen Entwicklungen der Präzisionsmedizin in der Kinderneurologie im Bereich Diagnostik, Prävention und zielgerichteter Therapien.

**Diskussion:**

Der Paradigmenwechsel in Folge der Präzisionsmedizin beruht auf einem stärker auf das Individuum und seinen einzigartigen Eigenschaften ausgerichteten Behandlungsansatz. Zur genauen Beschreibung und Charakterisierung der betroffenen Kinder werden die modernen Methoden der genetischen und molekularen Diagnostik eingesetzt, ergänzt durch eine genaue Beschreibung des klinischen Erscheinungsbildes. Dennoch ist der Erfolg der daraus abgeleiteten, individuell besten Behandlungsstrategie oft vom Zeitpunkt der Diagnosestellung abhängig. Daher rücken zunehmend Methoden zur Krankheitsprävention, insbesondere das Neugeborenenscreening, in den Vordergrund, um den bestmöglichen Erfolg der neuartigen Therapien bereits vor Ausbruch von Krankheitssymptomen zu erreichen. Neben einer präzisen Stratifizierung der Therapien sollte in Zukunft auch ein besonderes Augenmerk auf der Berücksichtigung der individuellen Perspektive der Patienten und Erziehungsberechtigten liegen. Darüber hinaus müssen für die sinnvolle Anwendung der genmodifizierenden Therapien in Deutschland qualitätsgesicherte Rahmenbedingungen geschaffen werden.

## Abstract (en)

**Background:**

In recent years the possibilities for molecular diagnostics and treatment of rare childhood diseases have greatly improved. The first gene-modifying drugs have now been approved, leading to a new era of precision treatment in pediatric neurology.

**Objective:**

This article describes the dynamic developments of precision medicine in pediatric neurology in the areas of prevention, diagnostics and targeted treatment.

**Discussion:**

The paradigm shift as a result of precision medicine is based on a treatment approach focused more strongly on the individual and the corresponding unique characteristics. Modern methods of genetic and molecular diagnostics are used to accurately describe and characterize affected children, complemented by a precise description of the clinical phenotype. Nevertheless, the success of the best individual treatment strategy derived from this information is often dependent on the time of diagnosis. Therefore, methods for disease prevention, particularly newborn screening programs, become increasingly more important to achieve the best possible success of novel therapies even before the onset of disease symptoms. In addition to a precise stratification of therapies, special attention should be paid in the future to the consideration of the individual perspective of patients and parents/guardians. Furthermore, a normative framework for a quality-ensured application of gene-modifying therapies in the German healthcare system must be created.

In der Vergangenheit richteten sich viele Behandlungen in der Kinderneurologie an der bestmöglichen Kontrolle bzw. Unterdrückung von Krankheitssymptomen aus, individuelle Unterschiede im Ansprechen und in der Verträglichkeit von Medikamenten wurden wenig berücksichtigt. Der Einsatz moderner diagnostischer Methoden zur maßgeschneiderten Krankheitsprävention und -behandlung erlaubt im besten Fall eine frühzeitigere bis präsymptomatische Diagnosestellung und in Zukunft eine gezieltere Stratifizierung im Hinblick auf die Definition von Krankheitssubgruppen zur besseren Vorhersage des individuellen Therapieansprechens.

## Definition und Begrifflichkeiten

**Figure 1:**
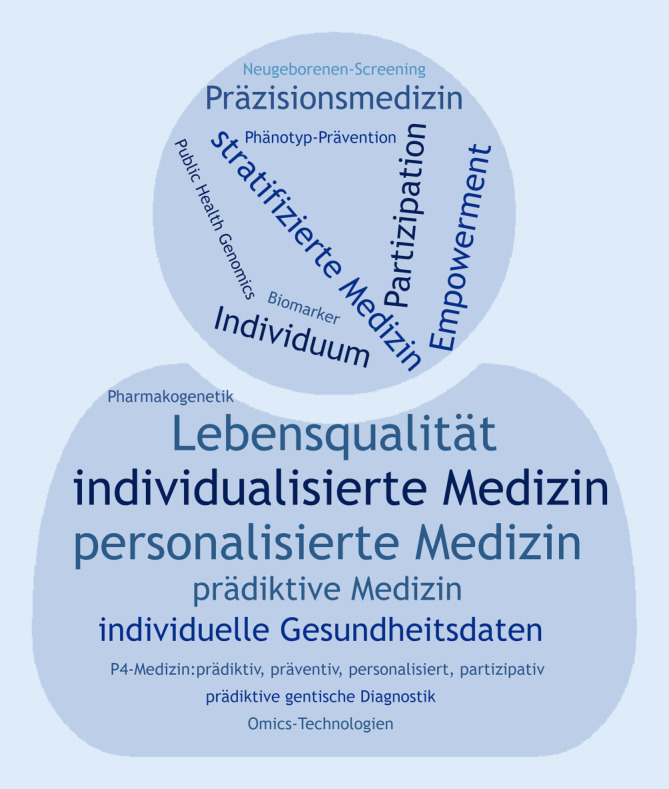

In den letzten Jahren haben sich die Möglichkeiten der molekularen Diagnostik seltener neurologischer und onkologischer Erkrankungen stark verbessert. Die Auswahl von Arzneimitteln zur gezielten Therapie dieser Störungen kann dadurch stärker auf das Individuum ausgerichtet werden. Erstmals können auf diese Weise angeborene genetische Erkrankungen in der Kinderneurologie gezielt und kausal behandelt werden. Häufig wird diese bahnbrechende Entwicklung als sog. Präzisionsmedizin zusammengefasst. Die genauen Ursprünge dieses Begriffes lassen sich nur schwer fassen, mehrere Begriffe zur Beschreibung dieses Ansatzes werden synonym und weitgehend unpräzise angewendet [2]. Abb. 1 fasst die in diesem Kontext verwendeten Begrifflichkeiten zusammen, die im Folgenden geordnet und auf die aktuellen Entwicklungen in der Kinderneurologie übertragen werden sollen.

Präzisionsmedizin ist die „richtige Behandlung zur richtigen Zeit beim richtigen Patienten“

Laut Definition der US Food and Drug Administration (FDA) versteht man unter dem Begriff *Präzisionsmedizin* (synonym als *personalisierte Medizin* bezeichnet) einen „innovativen Ansatz zur maßgeschneiderten Krankheitsprävention und -behandlung, der Unterschiede in den Genen, der Umwelt und dem Lebensstil der Menschen berücksichtigt“. Ziel der Präzisionsmedizin sei es, die „richtigen Behandlungen zur richtigen Zeit auf die richtigen Patienten“ auszurichten [36]. Im Jahr 2015 erfuhr dieser Ansatz durch die vom damaligen US-Präsidenten Obama in seiner Rede zur Lage der Nation initiierte Präzisionsmedizininitiative einen deutlichen Schub. Im Leitbild der Initiative wird auch die Vernetzung verschiedener komplexer Datensätze aus Genomsequenzen, Mikrobiomzusammensetzung, Anamnesedaten, Lebensstil und individuellen Ernährungsgewohnheiten zur Beschreibung und bestmöglichen Charakterisierung eines Individuums thematisiert, aus deren Informationen die individuell beste Präventions- und Behandlungsstrategie abgeleitet werden soll [35]. Gerade der Austausch und die Interoperabilität großer Datensätze (Big Data) unter Einhaltung des Datenschutzes wird im Rahmen der Initiative als wesentlicher Teil der Präzisionsmedizin angesehen. Neben der Interoperabilität spielen insbesondere die Auffindbarkeit, Zugänglichkeit und Wiederverwendbarkeit solcher Datensätze eine zentrale Rolle (sog. FAIR[„findable, accessible, interoperable, re-usable“]-Prinzipien; [39]).

In der Europäischen Union wird der Begriff der Präzisionsmedizin weniger häufig eingesetzt, es wird mehr von *personalisierter Medizin* gesprochen, deren Definition in den Schlussfolgerungen des Europäischen Rates zur personalisierten Medizin enthalten ist (2015/C 421/03):

Personalisierte Medizin bezieht sich auf ein medizinisches Modell, das die Charakterisierung des individuellen Phänotyps und Genotyps (z. B. molekulare Informationen, medizinische Bildgebung und Lebensstildaten) einsetzt, um spezifisch für jeden Menschen und zur richtigen Zeit maßgeschneiderte Behandlungsstrategien anzubieten und/oder die Prädisposition für Erkrankungen festzustellen und/oder zielgerichtet und rechtzeitig Präventionsansätze bereitzustellen [30].

**Figure 2:**
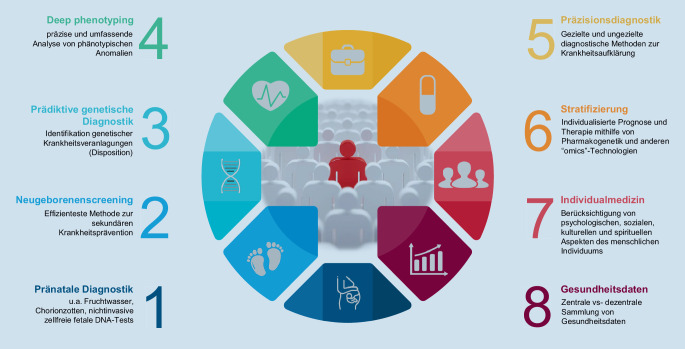

Deutschland folgt weitgehend den europäischen Definitionen [7], zusätzlich wird von der Bundeszentrale für gesundheitliche Aufklärung der Begriff der *prädiktiven Medizin* als Teilgebiet der *individualisierten Medizin* eingeführt, der als Synonym für die *personalisierte, stratifizierte* oder *maßgeschneiderte Medizin* verwendet wird und zu der auch die von Präsident Obama proklamierte *Präzisionsmedizin* gehört [19]. In Abb. 2 sind die wesentlichen Elemente der Präzisionsmedizin zusammengefasst. Während sich die v. a. in Großbritannien verwendete *stratifizierte Medizin* v. a. auf die individualisierte Therapie bezieht, kommt der Präzisionsmedizin eine deutlich weitreichendere Bedeutung zu, die ihren Ursprung in der sog. *P4-Medizin* findet (prädiktiv, präventiv, personalisiert und partizipativ; [15]).

Letztendlich ist allen genannten Begrifflichkeiten gemein, dass verschiedene Untergruppen von Krankheiten existieren, dass Menschen individuelle Krankheitsrisiken tragen und dass Medikamente oder andere Behandlungen nicht bei allen Patienten eine gleich gute Wirksamkeit und Sicherheit aufweisen. Ein weiterer gemeinsamer Ansatz ist eine stärkere Berücksichtigung individueller Unterschiede in der biologisch-genetischen Ausstattung bei der klinischen Entscheidungsfindung und Stratifizierung von Therapien. Übertragen auf die Medizin des Kindes- und Jugendalters können folgende wesentliche Elemente der im Folgenden weiterhin *Präzisionsmedizin* genannten individualisierten Diagnostik und Therapie beschrieben werden:

### Pränatale Diagnostik (1)

Neben den bekannten Methoden zur pränatalen Diagnosefindung (Fruchtwasseruntersuchung/Chorionzottenbiopsie) werden in den letzten Jahren immer weitreichendere Methoden zur Krankheitsaufklärung genutzt, z. B. die nichtinvasiven zellfreien fetalen DNA-Tests, bei denen fetale DNA aus dem mütterlichen Blut isoliert und anschließend weiterführend genetisch untersucht werden kann. Inzwischen können hier auch Einzel‑/Gruppengenanalysen durchgeführt werden [42]. In Zukunft ist denkbar, dass Screeninguntersuchungen in speziellen Risikokonstellationen während der Schwangerschaft durchgeführt und verfügbare Therapien, z. B. die Genersatztherapie, bereits intrauterin, z. B. über die Nabelschnurvene, appliziert werden.

### Neugeborenenscreening (2)

In Deutschland steht mit dem Neugeborenenscreening die wirksamste Methode zur Sekundärprävention von Erkrankungen zur Verfügung. Während der Ursprung der Neugeborenenscreeningprogramme in der Untersuchung diagnostischer Biomarker bei angeborenen Stoffwechselstörungen und endokrinologischen Erkrankungen lag und diese in den letzten Jahren stetig erweitert wurden [21], kamen seit Aufnahme der zystischen Fibrose (2016) in den Neugeborenenscreeningkanon auch genetische Screeningmethoden zum Einsatz und im Oktober 2021 wurden mit der Aufnahme der spinalen Muskelatrophie und Sichelzellanämie in das Screeningprogramm zum ersten Mal genetische Primärvariablen flächendeckend eingesetzt (siehe Beitrag von Kölbel et al. in dieser Ausgabe von *Der Nervenarzt* [18]). In Zukunft werden genetische Hochdurchsatzmethoden, insbesondere im Zeitalter zunehmend verfügbarer molekularer Therapien, eine große Rolle in der Weiterentwicklung des Neugeborenenscreenings spielen [41].

### Prädiktive genetische Diagnostik (3)

Ziel dieser oft breit angelegten genetischen Diagnostik ist die frühzeitige Identifikation einer individuellen Veranlagung zur Entwicklung von Krankheiten, die sich erst später im Leben manifestieren (z. B. Screening auf das *BRCA*-Gen bei familiärer Brustkrebsdisposition). Diese Form der Diagnostik spielt aber zunehmend auch in der Kinderneurologie eine wichtige Rolle. Nach dem Auftreten erster epileptischer Anfälle im Neugeborenen- bzw. Säuglingsalter werden inzwischen zunehmend genetische Hochdurchsatzsequenzierungen im Rahmen der bestmöglichen Prädiktion des klinischen Schweregrades der Epilepsie und zugleich zur Stratifikation einer möglichst zielgerichteten antikonvulsiven Therapie angewendet [20].

### „Deep phenotyping“ (4)

Präzisionsmedizin erfordert ein genaues Verständnis der Beziehung zwischen der gefundenen genetischen Variabilität und dem daraus resultierenden klinischen Phänotyp. Die klassischen klinischen Beschreibungen, z. B. in Arztbriefen, reichen dafür nicht aus, es ist eine wesentlich feingranulärere, auf interoperablen Datenwörterbüchern, z. B. der sog. Human Phenotype Ontology (HPO), basierende Beschreibung der Krankheitsmerkmale notwendig, was als „deep phenotyping“ bezeichnet wird [10]. Die differenzierte und HPO-basiert numerisch kodierbare Beschreibung kann als Grundlage für Genotyp-Metabotyp-Phänotyp-Analysen herangezogen werden.

### Präzisionsdiagnostik (5)

Neben den bereits erwähnten genetischen Hochdurchsatzverfahren, z. B. dem „next generation sequencing“, kommen in den letzten Jahren zunehmend auch neue Multikomponentenanalysen zum Einsatz, z. B. proteomische oder metabolomische Analysen [40]. Ihr Stellenwert liegt nicht nur in der Krankheitsaufklärung und Identifikation neuer Proteinzielstrukturen oder Metaboliten als Ziele für krankheitsmodifizierende Therapien, sondern auch in der longitudinalen Beurteilung des Therapieansprechens. In Zukunft erhofft man sich auch in der Kinderneurologie durch den breiten Einsatz dieser Technologien vor und nach Applikation einer krankheitsmodifizierenden Therapie ein besseres pathophysiologisches Verständnis und neue Therapiemarker für ein individuell unterschiedliches Therapieansprechen („Responder“ vs. „Nonresponder“).

### Stratifizierung (6)

Die bereits im Rahmen der Präzisionsmedizin dargestellte Einteilung in Krankheitssubgruppen und die bestmögliche Charakterisierung der Eigenschaften des jeweiligen Individuums stellen letztendlich die Grundlage für eine stratifizierte Therapie dar. In diesem Kontext wird bei der gezielten Auswahl der individuell besten Therapieoption oft der Begriff „targeted therapies“ – gezielte Therapien – z. B. bezogen auf eine spezifische Ionenkanalmutation bei Epilepsien, verwendet [9, 25]. Neben der oben dargestellten Charakterisierung des Ausgangszustandes werden heute zunehmend auch Methoden der Pharmakogenetik und anderen Omics-Technologien herangezogen, um eine individualisierte Prognose für das Therapieansprechen sowie das Risiko für das Auftreten einer bestimmten Nebenwirkung abschätzen zu können. Ein Beispiel für die stratifizierte Therapie ist die vom Genotyp abhängende gezielte Therapie mit Antisense-Oligonukleotiden (ASO), wie sie bei der Muskeldystrophie Duchenne zunehmend versucht wird.

### Individualmedizin (7)

Dieser sehr wichtige Bereich wird von einzelnen Autoren als wesentlicher konzeptioneller Unterschied zwischen den Begriffen *Präzisionsmedizin* und *personalisierte Medizin* gesehen. Nach der Beschreibung der Präzisionsmedizininitiative ist diese Unterscheidung aber nicht korrekt, sondern findet in beiden Konzepten Anwendung. Das Konzept der Individualmedizin fokussiert besonders auf die Berücksichtigung sozialer, kultureller und spiritueller Unterschiede des menschlichen Individuums und seiner Umgebung, die im Rahmen der Präzisionsdiagnostik und -therapie unbedingt berücksichtigt werden müssen.

### Gesundheitsdaten (8)

Es existieren zahlreiche nationale und internationale Initiativen und Netzwerke, um den Austausch der im Rahmen der Präzisionsmedizin gewonnenen Gesundheitsdaten zu fördern [26]. Eine wesentliche Voraussetzung ist die bereits erwähnte Interoperabilität der Datensätze und deren Lesbarkeit in unterschiedlichen Datenbankformaten. Von der ursprünglich oft kolportierten Datenhaltung in zentralen Krankheitsregistern wird in den letzten Jahren zunehmend Abstand genommen, eine lokale Datenhaltung unter Einhaltung und Gewährleistung einer semantischen und syntaktischen Interoperabilität mit der Möglichkeit zum Datenaustausch und der standardisierten Auswertung in Forschungsnetzwerken wird dabei als ein Modell für die Zukunft gesehen [24].

## Präzisionstherapie in der Kinderneurologie

**Figure 3:**
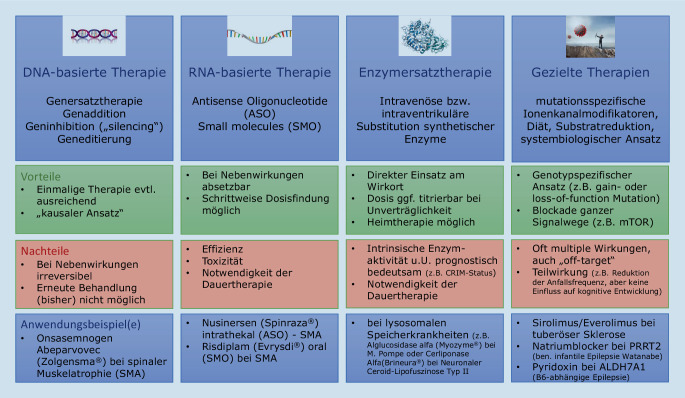

Neben der Onkologie kann die Kinderheilkunde und v. a. die Kinderneurologie als interdisziplinär agierendes Fach als Schrittmacher für die Präzisionsmedizin der Zukunft betrachtet werden [33]. Die Mehrzahl angeborener genetischer Erkrankungen mit Beteiligung des zentralen und peripheren Nervensystems sowie der quergestreiften Skelettmuskulatur manifestiert sich im Verlauf des Kindes- und Jugendalters, Präzisionstherapie setzt demnach bereits ab dem Neugeborenenalter an, gerade wenn sie in Zukunft präventiv, d. h. vor der klinischen Manifestation einer neurologischen Erkrankung eingesetzt werden soll. Grundsätzlich unterscheidet man je nach Wirkansatz verschiedene Gruppen von Präzisionstherapeutika, die in Abb. 3 mit wesentlichen Vor- und Nachteilen sowie klinischen Anwendungsbeispielen zusammengefasst sind.

### DNA-basierte Gentherapien

DNA-basierte Therapien setzen weitgehend „kausal“, d. h. auf der Ebene der krankheitsverursachenden genetischen Veränderung an. Sie sind Teil einer auch als Arzneimittel für neuartige Therapien, „advanced therapeutic medicinal products“ (ATMPs), bezeichneten Gruppe von Präzisionstherapeutika. ATMPs umfassen Gentherapeutika, somatische Zelltherapeutika und biotechnologisch behandelte Gewebeprodukte. Sie verändern die Therapielandschaft grundlegend, da sie als Einmalgabe das Potenzial haben, den Krankheitsverlauf signifikant und nachhaltig zu verbessern bzw. im besten Fall dauerhaft zu heilen [11]. Im Gegensatz zu traditionellen Medikamenten, die patientenübergreifend eingesetzt werden, sind ATMPs individualisierte Therapien, die gewichtsbasiert für einzelne oder für kleine Gruppen von Kindern hergestellt werden. Die Transduktion und damit das Einbringen des fremden Gens (Transgen) in den menschlichen Organismus erfolgt bei der klassischen Gentherapie über nichthumanpathogene virale Vektoren, in die das Transgen vor Applikation eingebracht wird, und die quasi als „Shuttle-Busse“ funktionieren.

Für Gentherapien neuromuskulärer Erkrankungen eignen sich besonders adenoassoziierte Viren

Während verschiedene virale Vektoren für gentherapeutische Ansätze getestet wurden, darunter lentivirusbasierte Therapien für einige Leukodystrophien [5], haben sich für Gentherapien neuromuskulärer Erkrankungen besonders adenoassoziierte Viren (AAV) bewährt [1]. Die Auswahl des AAV-Serotyps erlaubt aufgrund unterschiedlicher Gewebeaffinitäten (Tropismus) die gezielte Ansteuerung spezifischer Organsysteme bzw. Zielzellen. So eignet sich der Serotyp AAV9 durch die Fähigkeit, die Blut-Hirn-Schranke zu überwinden, gut für eine minimal-invasive, intravenöse Applikation bei neurologischen Erkrankungen.

**Figure 4:**
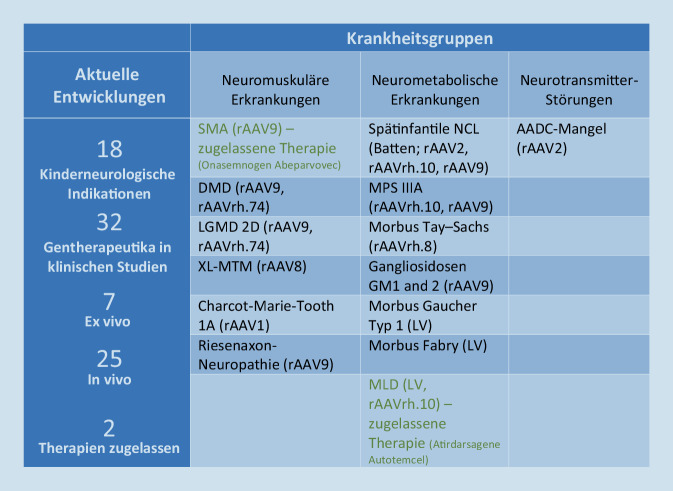

Einen Überblick über die aktuell in der Kinderneurologie in Entwicklung befindlichen und bereits zugelassenen Gentherapeutika und die für ihre erfolgreiche Transduktion in die Zielzelle verwendeten Vektoren gibt Abb. 4. Klinisch angewandt werden ATMPs derzeit in der Kinderneurologie u. a. als In-vivo-Genersatztherapie mit dem Präparat Onasemnogen Abeparvovec (Handelsname: Zolgensma®) zur Behandlung von Neugeborenen und Kleinkindern mit spinaler Muskelatrophie (SMA; [43]), d. h. das zu ersetzende Gen wird in Form eines in ein AAV9-Viruskapsid eingebrachtes Transgen direkt intravenös appliziert. Im Dezember 2020 wurde darüber hinaus mit Libmeldy® (Atidarsagen Autotemcel) eine erste Ex-vivo-Gentherapie zur Behandlung der metachromatischen Leukodystrophie (MLD) zugelassen. Diese Gentherapie enthält eine mit autologen CD34^+^-Zellen angereicherte Population hämatopoetischer Stamm- und Vorläuferzellen, die ex vivo lentiviraltransduzierte Kopien des Arylsulfatase-A-Gens enthalten. Die Zellen werden vor der Therapie und nach Konditionierung per Leukapherese gewonnen, gentherapeutisch behandelt und anschließend den Kindern autolog appliziert [12]. In Zukunft wird auch mit der Zulassung der ersten intrathekal zu applizierenden Gentherapie zur Therapie des sog. AADC-Mangels (OMIM 608643), einer Neurotransmitterstörung, gerechnet. Die einmalige Applikation der AAV2-basierten Gentherapie erfolgt dabei direkt intrazerebral im Rahmen einer stereotaktischen neurochirurgischen Operation mit bilateraler Platzierung zweier Kanülen in das Putamen oder die Substantia nigra [23].

**Figure 5:**
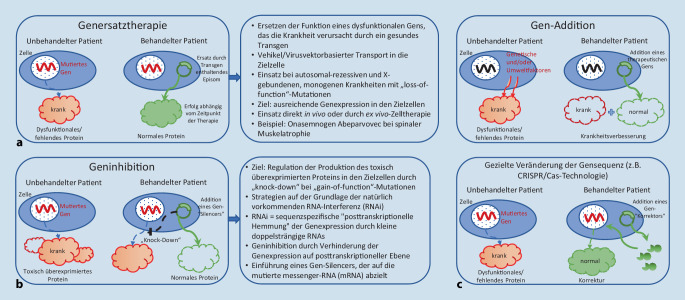

In Abb. 5 sind die wesentlichen Prinzipien dargestellt, die aktuell in der Entwicklung neuer Gentherapeutika zum Einsatz kommen. In der klinischen Anwendung befinden sich zurzeit vorwiegend „additive Gentherapien“ (Abb. 5a). Ein weiteres wichtiges Zukunftsprinzip der Gentherapien ist eine gentherapeutische Modifikation auf Ebene der RNA-Transkription mit dem Ziel der Regulation der Produktion eines toxisch-überexprimierten Gens, z. B. bei „Gain-of-function“-Mutationen, der sog. Geninhibition oder auch „Knock-down“-Gentherapie. Dieser Ansatz basiert auf der Grundlage der auch natürlich vorkommenden RNA-Interferenz (RNAi), eine sequenzspezifische, posttranskriptionelle Hemmung der Genexpression durch kleine, doppelsträngige RNAs. Erste Ansätze für geninhibitorische Therapien in der Kinderneurologie befinden sich bereits in frühen Phasen klinischer Studien [14].

Ebenfalls an der Schwelle zum Übergang in die klinische Erstanwendung an betroffenen Kindern ist die Geneditierung (Abb. 5c), bei der einzelne Gensequenzen gezielt verändert werden mit dem Ziel, die ursprüngliche Gensequenz eines mutierten Gens oder Genabschnitts wieder herzustellen. Grundlage der Geneditierung ist z. B. das sog. CRISPR-System („clustered regularly interspaced short palindromic repeats“), welches Abschnitte sich wiederholender DNA („repeats“) enthält, die ursprünglich im Erbgut vieler Bakterien zu finden sind. Die Bakterien bedienen sich einem gezielten Mechanismus, dem sog. CRISPR/Cas-System, für die Abwehr und Resistenzbildung gegenüber fremdem Erbgut von Viren oder Plasmiden. Das CRISPR/Cas-System wird als Grundlage für viele Geneditierungsmodelle angewendet. Aktuell spielen v. a. Off-target-Mechanismen noch eine einschränkende Rolle, die zu unerwünschten Wirkungen und Strangbrüchen abseits des eigentlichen DNA-Zielabschnitts führen [3]. Es ist damit zu rechnen, dass in den kommenden Jahren zunehmend auch geninhibitorische und Geneditierungsansätze in die frühen Phasen klinischer Studien in der Kinderneurologie kommen werden.

### RNA-basierte Therapien

Der Erfolg der Präzionstherapie in der Kinder- und Erwachsenenneurologie in den letzten Jahren basiert maßgeblich auf diesem Ansatz, dessen Ziel eine Veränderung der Expression des Zielproteins durch Ansatz auf Ebene der Boten-RNA (Messenger-RNA, mRNA) darstellt. Die beiden wichtigsten therapeutischen Ansätze auf diesem Gebiet sind die Antisense-Oligonukleotide (ASOs), die die mRNA-Translation hemmen, und die Oligonukleotide, die über den RNA-Interferenzweg funktionieren (sog. „interfering RNA“; [8]). Um die Zielgenexpression zu regulieren, müssen diese Verbindungen krankheitsassoziierte Gewebe und Zellmembranen erreichen.

ASOs setzen an der Herunter- oder Hochregulierung molekularer Zielstrukturen an

Ein ASO ist ein einzelsträngiges Desoxyribonukleotid, dass an das mRNA-Target bindet mit dem Ziel, durch unterschiedliche Mechanismen die Herunter- oder Hochregulierung einer molekularen Zielstruktur zu erreichen. Dazu gehört u. a. die Veränderung des Spleißprozesses (Splicing-Modifikation), ein Ansatz, der zur Therapie der spinalen Muskelatrophie (SMA) eingesetzt wird. Pathophysiologisch ist die SMA charakterisiert durch homozygote Deletionen oder Loss-of-function-Mutationen des Survival-motor-neuron-1(*SMN1*)-Gens, mit Restfunktion einer variablen Kopienzahl eines paralogen *SMN2*-Gens, welches jeweils nur unzureichende Mengen an funktionalem SMN-Protein produziert. Das im Juli 2017 für die Therapie der SMA zugelassene ASO Nusinersen (Handelsname Spinraza®) setzt über diesen alternativen SMN-2-Weg an, bei dem im natürlichen Prozess auf Ebene der mRNA-Prozessierung das Exon 7 des *SMN2*-Gens entfernt wird, was zu einem instabilen, rasch zugrunde gehendem SMN-Protein führt. Durch gezielte Bindung von Nusinersen an den intronisch liegenden „splice site silencer 1“ (ISS-1) wird Exon 7 nicht länger im Rahmen des Splicing-Vorgangs entfernt und es entsteht stabiles, funktionales SMN-Protein.

Einen ganz ähnlichen Ansatz verfolgt das im April 2021 zugelassene Präparat Risdiplam (Handelname Evrysdi®), welches den gleichen Effekt wie Nusinersen über eine Bindung an den exonischen Splicing-Enhancer 2 (ESE-2) erreicht. Im Unterschied zu Nusinersen kann Risdiplam aufgrund seiner kleineren Molekülstruktur als sog. „small molecule“ oral appliziert werden, weil es über die Blut-Hirn-Schranke direkt ins Zentralnervensystem und auch in alle peripheren Organe diffundieren kann.

Auch bei der Muskeldystrophie Typ Duchenne und anderen Erkrankungen in der Kinderneurologie befinden sich zahlreiche ASOs in unterschiedlichen Phasen der klinischen Entwicklung, ihnen allen ist gemein, dass sie wiederholt und dauerhaft appliziert werden müssen [13].

### Enzymersatztherapien

Die Enzymersatztherapien („enzyme replacement therapy“, ERT) kommen bereits seit einigen Jahren bei lysosomalen Speichererkrankungen zum Einsatz. Diese gehen mit einem genetisch determinierten Enzymdefekt einher, der zu einem Mangel des jeweiligen Enzyms mit der Folge einer Akkumulation von Makromolekülen in unterschiedlichen Geweben führt. Bei der ERT werden rekombinante humane Enzyme in regelmäßigen Abständen per infusionem appliziert. Mit der Enzymersatztherapie können nur die diejenigen Symptome der lysosomalen Speicherkrankheiten therapiert werden, die nicht das zentrale Nervensystem betreffen, ähnlich wie bei den ASOs ist die Größe der Moleküle ein limitierender Faktor, um erfolgreich die Blut-Hirn-Schranke passieren zu können. Ein sehr vielversprechender Ansatz ist die direkt intrathekal zur Anwendung kommende und inzwischen zugelassene Enzymersatztherapie der spätinfantilen neuronalen Ceroid-Lipofuszinose Typ II mit Cerliponase Alfa (Brineura), deren Wirksamkeit erfolgreich nachgewiesen werden konnte [32].

### Gezielte Therapien

Unter den gezielten Therapien („targeted therapies“) kann eine Reihe präzisionstherapeutischer Ansätze zusammengefasst werden, deren Gemeinsamkeit darin besteht, dass sie die Folgen genetisch determinierter Erkrankungen direkt oder indirekt adressieren und oft erkrankungsspezifisch oder erkrankungsgruppenspezifisch eingesetzt werden. Beispiele für den Einsatz gezielter Therapien in der Kinderneurologie finden sich u. a. in der Epileptologie, wo zunehmend mutationsspezifische Ionenkanalmodifikatoren, z. B. bei frühkindlichen epileptischen Enzephalopathien, zum Einsatz kommen [22, 34]. Darüber hinaus werden spezielle Diäten eingesetzt, z. B. die ketogene Diät beim Glukosetransporterdefekt (GLUT1-Mangel), bei dem der Glukosetransport über die Blut-Hirn-Schranke gestört ist [17]. Andere Beispiele für gezielte Therapien in der Kinderneurologie sind die v. a. bei lysosomalen Speichererkrankungen zum Einsatz kommenden Substratreduktionstherapien [27] oder systembiologische Ansätze wie die Therapie mit den mTOR(„mechanistic target of Rapamycin“)-Inhibitoren Everolimus bzw. Sirolimus bei tuberöser Sklerose [31].

## Therapieerfolg abhängig vom Zeitpunkt

**Figure 6:**
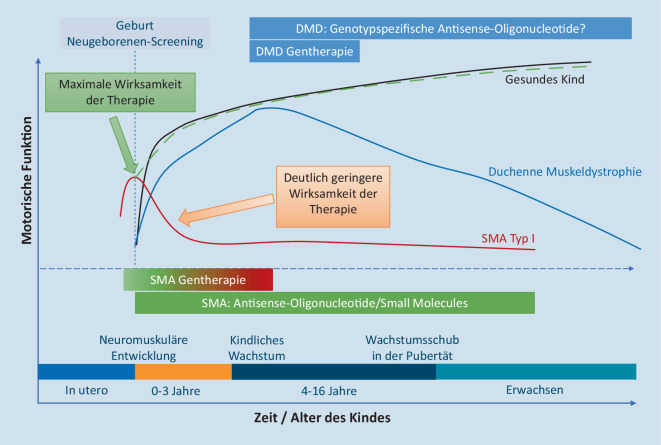

Ein ganz wesentliches Prinzip, das für die meisten präzisionstherapeutischen Ansätze gilt, ist die Abhängigkeit des Therapieerfolges vom Zeitpunkt des Behandlungsbeginns. Grundsätzlich gilt, je weiter eine Erkrankung zum Therapiezeitpunkt fortgeschritten ist und die daraus resultierenden Folgen bereits manifestiert sind, desto schlechter wird der Erfolg der Präzisionstherapie sein, unabhängig vom gewählten Wirkansatz. Abb. 6 illustriert dieses Grundprinzip am Beispiel der neuen Therapieansätze für die neuromuskulären Erkrankungen SMA und die Duchenne Muskeldystrophie (DMD).

Bei Kindern mit spinaler Muskelatrophie Typ I, die in der Mehrzahl 2 SMN-2-Kopien aufweisen, gehen oft bereits im letzten Schwangerschaftstrimenon Motoneuronen zugrunde, weil der Bedarf an SMN-Protein bereits ab der 32. Schwangerschaftswoche im Rahmen der Synaptogenese stark zunimmt. Ein erheblicher Teil der Kinder mit früher Manifestation der Erkrankung in den ersten Lebensmonaten weißt daher bereits bei der Geburt Symptome der Erkrankung auf [37]. Die maximale Wirksamkeit einer krankheitsmodifizierenden Therapie, unabhängig vom Wirkansatz, besteht ganz eindeutig vor Beginn der Phase der Neurodegeneration mit zunehmendem Verlust an Motorneuronen. Die Einführung des flächendeckenden Neugeborenenscreenings auf SMA in Deutschland ab Herbst 2021 wird die Entwicklung neugeborener Kinder mit SMA daher bereits erheblich positiv beeinflussen. Bei Kindern mit spinaler Muskelatrophie Typ I und 2 SMN2-Kopien ist aber trotz Neugeborenenscreenings und sofortigen Therapiebeginns zwar mit einer erheblich verbesserten, aber dennoch dauerhaft eingeschränkten Entwicklung der motorischen Funktionen zu rechnen.

Bei DMD setzen gentherapeutische Ansätze überwiegend zwischen dem 4. bis 7. Lebensjahr an

Ob es Unterschiede im Ansprechen zwischen den drei zur Verfügung stehenden Präzisionstherapeutika für die SMA gibt, kann lediglich die genaue klinische Verlaufsbeobachtung in großen Krankheitsregistern, die Real-world-Evidenz, beantworten. Klinische „Head-to-head“-Vergleichsstudien sind bei den Orphan-Therapeutika nicht grundlegende Bedingung der Zulassung und damit meist zum Zeitpunkt der Marktzulassung nicht vorhanden.

Bei der Duchenne Muskeldystrophie stellt sich die Situation exemplarisch ganz anders dar. Die Kinder mit DMD zeigen eine allenfalls leicht verzögerte frühkindliche motorische Entwicklung, etwa bis zum Vorschulalter gewinnen die Jungen mit DMD noch an motorischen Fähigkeiten hinzu, bevor nach einer kurzen Plateauphase der zunehmende Verlust an motorischen Funktionen einsetzt. Aktuelle gentherapeutische Ansätze setzen daher überwiegend in dieser Plateauphase am Zenit der motorischen Entwicklung, etwa zwischen dem 4. bis 7. Lebensjahr, an um den bestmöglichen Effekt der Therapie zu erreichen [16]. Da die Phase der Neurodegeneration zeitlich sehr variabel und auch interindividuell sehr unterschiedlich einsetzt, kann der Erfolg einer krankheitsmodifizierenden DMD-Gentherapie in den klinischen Studien deutlich schwerer und v. a. nur langfristiger gut beurteilt werden. Dieser Umstand ist für eine schnelle Zulassung der Präparate deutlich erschwerend, weil die klassischen Beobachtungszeiträume in klinischen Studien maximal 24 Monate zum Erreichen des primären Endpunkts betragen, bei DMD ein zu kurzer Zeitraum zur Beurteilung einer eindeutigen Veränderung der Krankheitstrajektorien. Darüber hinaus kommt bei den gentherapeutischen Ansätzen der quergestreiften Muskulatur hinzu, dass es sich im Vergleich zu den postmitotischen motorischen Vorderhornzellen bei der SMA bei der Skelettmuskulatur um ein sehr teilungsaktives Gewebe handelt, wo möglicherweise eine zunehmende „Verdünnung“ des Effekts durch Zellteilung und Regeneration von Zellen aus muskulären Vorläuferzellen, den sog. Progenitorzellen, stattfindet. Der Erfolg der Therapieansätze bei der DMD bleibt also abzuwarten [4].

## Unzureichende Rahmenbedingungen in Deutschland

In Deutschland existieren bisher keine ausreichend guten Rahmenbedingungen für die Anwendung der innovativen und teuren Präzisionstherapeutika [29]. Die komplexe Diagnostik, interdisziplinäre Betreuung und Therapie der Kinder können nur in ausreichend spezialisierten Kompetenzzentren, z. B. den Zentren für seltene Erkrankungen an den Universitätskliniken, erfolgen. Aktuell existieren zwar vom Gemeinsamen Bundesausschuss (G-BA) vorgegebene, hohe Qualitätsauflagen für die Anwendung und Nachsorge der neuen Therapien, eine ausreichende und kostendeckende finanzielle Ausstattung der Zentren fehlt aber bis dato. Neue und für alle Anwender einheitliche Vergütungspauschalen müssen transparent entwickelt und in der Versorgungsforschung getestet werden. Ansonsten wird der bahnbrechende und zukunftsweisende Erfolg der Präzisionsmedizin in der Kinderneurologie erheblich gefährdet, die Zentren können sich die Vorhaltung der notwendigen Ressourcen schlichtweg nicht mehr leisten.

## Fazit für die Praxis

- Die Kinderneurologie ist einer der Schrittmacher für die Präzisionsmedizin der Zukunft.
- Zahlreiche Präzisionstherapeutika mit unterschiedlichen Wirkansätzen sowie Vor- und Nachteilen befinden sich in unterschiedlichen Phasen der klinischen Entwicklung bzw. haben die Routineversorgung erreicht.
- Der Erfolg der neuen Therapien hängt in entscheidendem Maß vom Zeitpunkt der Diagnosestellung und einem frühzeitigen Therapiebeginn ab.
- Die Weiterentwicklung des nationalen Neugeborenenscreeningprogramms stellt die wirksamste Methode zur Sekundärprävention seltener, zumeist genetisch bedingter Erkrankungen dar.
- Datenerfassung, -format, -haltung und -auswertung spielen im Kontext der seltenen Erkrankungen und der Beurteilung sowie dem Vergleich von Wirksamkeit verschiedener Therapeutika eine zentrale Rolle.
- In Deutschland müssen optimierte und qualitätsgesicherte Rahmenbedingungen für die Anwendung und Nachsorge der komplexen neuen Therapien in spezialisierten Kompetenzzentren geschaffen werden.
